# Aggrecanopathy as an Underrecognized Cause of Idiopathic Short Stature: The Importance of Early Genetic Confirmation for Timely Diagnosis and Management—Case Reports and Literature Review

**DOI:** 10.3390/diseases14040127

**Published:** 2026-04-01

**Authors:** Aleksandra Sosin, Tetiana Tkachuk, Aleksandra Furtak, Magdalena Janeczko, Karol Stożek, Teofila Książek, Helena Poławska, Damian Loska, Sebastian Wardak, Jerzy Starzyk, Dominika Januś

**Affiliations:** 1 Students’ Scientific Group of Pediatric Auxology, Faculty of Medicine, University Children’s Hospital, Jagiellonian University Medical College, 31-008 Krakow, Poland; a.sosin@student.uj.edu.pl (A.S.); tetiana.tkachuk@student.uj.edu.pl (T.T.); 2 Department of Pediatric and Adolescent Endocrinology, University Children’s Hospital, 30-663 Krakow, Poland; aleksandra.furtak@uj.edu.pl (A.F.); jerzy.starzyk@uj.edu.pl (J.S.); 3 Department of Pediatric and Adolescent Endocrinology, Chair of Pediatrics, Institute of Pediatrics, Jagiellonian University Medical College, 31-008 Krakow, Poland; 4 Department of Medical Genetics, Faculty of Medicine, Jagiellonian University Medical College, 31-008 Krakow, Poland; magdalena.janeczko@uj.edu.pl (M.J.); teofila.ksiazek@uj.edu.pl (T.K.); 5 Laboratory of Cytogenetics and Molecular Genetics, University Children’s Hospital, 30-663 Krakow, Poland; karol.stozek@usdk.pl; 6 MedGen Medical Center, 02-954 Warsaw, Poland; helena.polawska@medgen.pl (H.P.); damian.loska@medgen.pl (D.L.); sebastian.wardak@medgen.pl (S.W.)

**Keywords:** short stature, *ACAN* gene, aggrecanopathy, advanced bone age, facial dysmorphism

## Abstract

**Background:** Short stature is a frequent clinical problem with a broad differential diagnosis. Emerging evidence indicates that pathogenic variants in the *ACAN* gene represent an underrecognized cause of growth failure and are often misclassified as idiopathic short stature. **Case presentation:** We report two pediatric patients harboring pathogenic *ACAN* gene variants, both presenting with short stature and distinctive facial dysmorphism. The first patient, a 15-year-old boy, exhibited short stature, advanced bone age, and a characteristic facial gestalt, including ptosis, hypertelorism, down-slanting palpebral fissures, and fleshy auricles, features not previously described in association with aggrecanopathy. Genetic analysis revealed a novel heterozygous frameshift variant, c.5677_5684del (p.Glu1893TrpfsTer8), in exon 12 of the *ACAN* gene. The second patient, a 5.5-year-old girl, presented with short stature, mild facial dysmorphism (down-slanting palpebral fissures and retracted mandible), and feeding difficulties. Copy number variation analysis identified a heterozygous deletion encompassing exons 15–19 of the *ACAN* gene. In both patients, the endocrine evaluation was unremarkable, and no chronic systemic disease was identified. The genetic findings were concordant with the clinical phenotype, confirming aggrecanopathy as the underlying cause of growth failure. **Conclusions:** These cases further delineate the phenotypic spectrum of *ACAN*-related short stature and underscore the diagnostic value of genetic testing in children with unexplained or idiopathic growth failure. Importantly, we expand the dysmorphological phenotype of aggrecanopathy by describing previously unreported facial features, which may facilitate earlier clinical recognition and diagnosis. The timely identification of pathogenic variants in the *ACAN* gene may have significant implications for patient management and long-term outcomes.

## 1. Introduction

Short stature is a common pediatric condition, defined as a height below (-) 2 standard deviation scores (SDS) for age and sex, and affects approximately 3% of the general pediatric population [1]. It is among the most frequent reasons for referral to pediatric endocrinology clinics. While nutritional deficiencies, chronic systemic diseases, endocrine disorders, chromosomal aberrations (e.g., trisomy 21 and Turner syndrome), and genetic syndromes (e.g., Noonan syndrome) account for a subset of cases, a substantial proportion of children has ultimately been classified so far as having idiopathic short stature (ISS) following a standard clinical evaluation [2,3].

Advances in molecular genetics have fundamentally changed the understanding of growth failure, revealing it to be a highly heterogeneous condition. Pathogenic variants have been identified in genes involved in growth plate biology, skeletal development, and cartilage homeostasis [4]. Among the monogenic causes of ISS, SHOX haploinsufficiency is the most common, accounting for approximately 2–3% of cases [4]. More recently, heterozygous pathogenic variants in the gene encoding aggrecan (*ACAN*) have emerged as an important and previously underrecognized cause of ISS, with the reported prevalence ranging from 1.4% to 6% in the unselected ISS cohorts [1,5,6,7,8,9,10,11].

**Figure 1:**
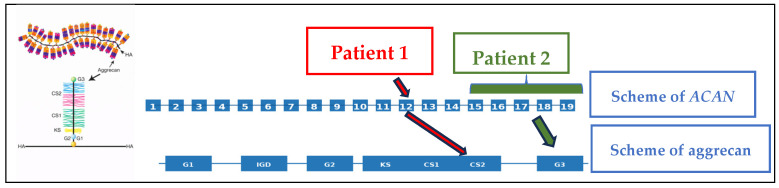
Structure of *ACAN* gene exons corresponding to aggrecan protein (right side) and structure of aggrecan attached to hyaluronian (HA) in extracellular matrix (left side) modified by Roughley and Mort [18] and Trigui et al. [3]. In Patient 1, the pathogenic variant in exon 12 affects the CS domain of aggrecan (red arrows), whereas, in Patient 2, the deletion of exons 15–19 disrupts the G3 domain (green arrows).

The human *ACAN* gene (OMIM: *155760) is located on chromosome 15q26.1 and consists of 19 exons encoding the full-length aggrecan core protein (Figure 1) [12,13]. With the increasing use of next-generation sequencing (NGS) in molecular diagnostics, heterozygous pathogenic variants in *ACAN* have been recognized as a significant cause of short stature and related skeletal phenotypes [1,2,3,4,5,6,7,8,9,10,11,12,13,14,15]. Pathogenic variants have been reported throughout the gene, affecting the distinct functional domains of the protein and resulting in a broad phenotypic spectrum collectively referred to as aggrecanopathies [7,14,15,16,17].

Aggrecan is a large chondroitin sulfate proteoglycan and a major structural component of the extracellular matrix of cartilage, including growth plate, articular, and intervertebral disc cartilage (Figure 1) [19,20]. The aggrecan core protein is composed of three globular domains (G1, G2, and G3), separated by an interglobular domain and a centrally located glycosaminoglycan (GAG) attachment region containing keratan sulfate and chondroitin sulfate chains [19,20] (Figure 1). The G1 domain mediates binding to hyaluronan and link protein, enabling the formation of large proteoglycan aggregates essential for cartilage integrity and load-bearing capacity [21]. Although the precise function of the G2 domain remains unclear, the C-terminal G3 domain—containing epidermal growth factor-like repeats, a complement regulatory protein-like domain, and a C-type lectin domain—interacts with extracellular matrix proteins such as tenascins and fibulins, contributing to the matrix assembly and stability [7,19,20]. The highly negatively charged GAG-rich region confers cartilage hydration and resistance to compressive forces, which are critical for normal longitudinal bone growth and joint function [22]. Beyond its biomechanical role, aggrecan regulates the availability and distribution of growth factors and signaling molecules within the cartilage extracellular matrix, thereby influencing chondrocyte differentiation, endochondral ossification, and bone morphogenesis [23].

Consistent with these domain-specific functions, pathogenic *ACAN* variants give rise to a continuum of skeletal phenotypes. Homozygous or compound heterozygous variants result in severe skeletal dysplasias, including autosomal recessive spondyloepimetaphyseal dysplasia, aggrecan type, SEMDAG (OMIM: #612813), and autosomal dominant spondyloepiphyseal dysplasia, Kimberley type, SEDK (OMIM: #608361) [24,25]. In contrast, heterozygous loss-of-function variants typically cause autosomal dominant short stature with variable expressivity, referred to as SSOAOD—short stature and advanced bone age with or without early-onset osteoarthritis and/or osteochondritis dissecans (OMIM: #165800) [7,14,21,23,26,27,28].

The most characteristic feature of autosomal dominant SSOAOD is proportionate short stature, frequently accompanied by advanced bone age, early growth cessation, and reduced adult height [7,14,21,23,26,27,28]. In contrast, disproportionate short stature has been observed in autosomal recessive SEMDAG [29]. Additional manifestations may include early-onset osteoarthritis, osteochondritis dissecans, intervertebral disc disease, and variable skeletal abnormalities. Although advanced bone age is considered a hallmark of heterozygous *ACAN*-related short stature, delayed or normal bone age has also been reported, underscoring the marked phenotypic heterogeneity of this condition [3]. Facial dysmorphic features have been described in a subset of individuals with *ACAN* variants; however, they are neither universal nor well-delineated [30]. Some features, including midfacial hypoplasia, may overlap with those observed in growth hormone deficiency, while others are more typically associated with genetic syndromes and are therefore not specific to aggrecanopathy [28,29].

Despite the growing recognition of *ACAN*-related growth disorders, aggrecanopathy remains underdiagnosed, particularly in children who lack overt skeletal dysplasia or endocrine abnormalities. A delayed diagnosis may result in missed opportunities for growth-promoting interventions and suboptimal adult height outcomes.

The aim of this study is to expand the clinical and dysmorphological spectrum of aggrecanopathy and to highlight the therapeutic implications of an early versus delayed diagnosis. We present two pediatric patients with pathogenic *ACAN* variants: a late-diagnosed adolescent boy with untreated aggrecanopathy, normal endocrine evaluation, and established short stature; and an early-diagnosed young girl in whom the timely recognition of the genetic etiology—guided by diagnostic pitfalls identified in the first patient— may allow the implementation of appropriate management strategies aimed at preventing future growth failure. By contrasting these two clinical trajectories, we underscore the importance of early genetic testing in children with unexplained short stature and demonstrate how the prompt diagnosis of aggrecanopathy may directly influence clinical decision-making and long-term growth outcomes.

## 2. Case Reports

### 2.1. Patient 1

The male patient was first noted to have a short stature at 11 years of age during a routine evaluation by a primary care physician and was subsequently referred for endocrine assessment. At presentation, his height was 135.6 cm (−2.0 SDS), with a body mass index (BMI) of 21.5 kg/m^2^. The mother’s height was 153 cm, whereas the father’s height was 182 cm, yielding a mid-parental height (MPH) of 174 cm corresponding to (-) 0.8 SDS.

The patient was born from the second pregnancy and second delivery at 39 weeks of gestation. The pregnancy was complicated by lower-extremity edema. The birth parameters were appropriate for gestational age, with a birth weight of 3660 g, length of 56 cm, and head circumference of 34 cm. The Apgar scores were 10 at both 1 and 5 min. The neonatal course was unremarkable, and psychomotor development proceeded within normal limits. There was no evidence of intellectual disability.

A physical examination revealed subtle but consistent dysmorphic features, including mild bilateral ptosis of the upper eyelids, hypertelorism, down-slanting palpebral fissures, fleshy auricles, and retrognathia. Additional findings included a mild limitation in elbow extension and flat valgus feet. Notably, similar facial features were observed in the patient’s mother. An anthropometric assessment demonstrated an increased arm span (171 cm)-to-height (160 cm) ratio (AS/H = 1.1), exceeding the age- and sex-specific reference values reported by Gerver et al. [31].

**Figure 2:**
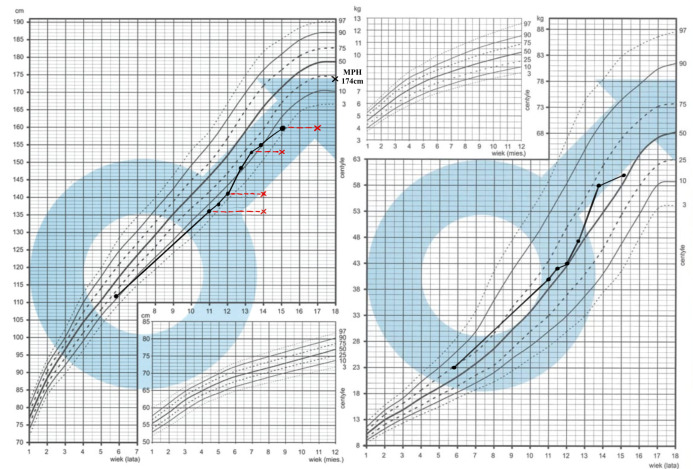
Growth charts of Patient 1. The Polish national charts were obtained from the following website: https://www.mp.pl/pediatria/praktyka-kliniczna/procedury/13848,ocena-rozwoju-somatycznego-dzieci-i-mlodziezy,1 (accessed on 12 December 2025, access after registration to Medycyna Praktyczna). Black dots present the measurements of height/weight. Red lines and red crosses correspond to bone age assessments.

**Figure 3:**
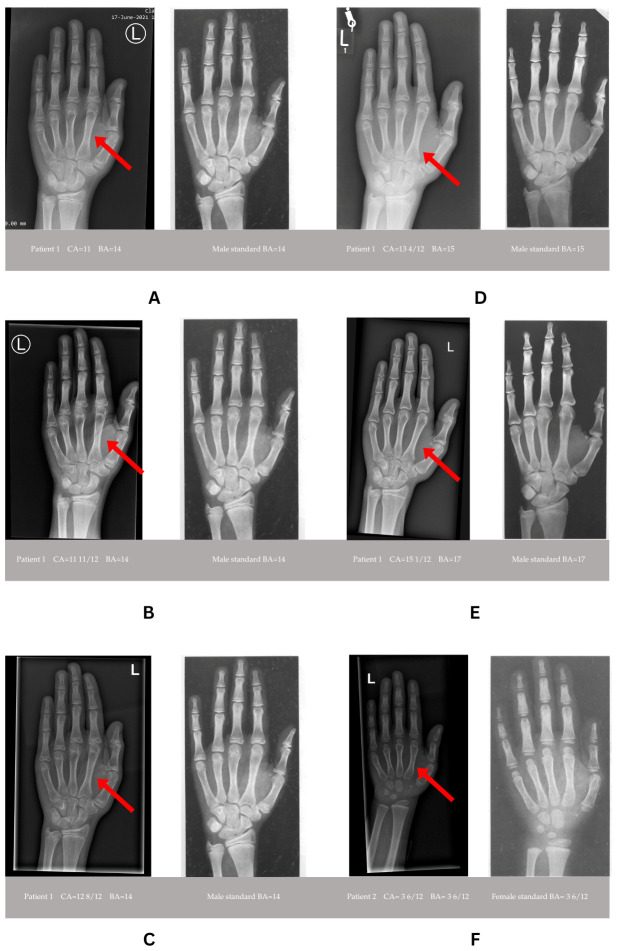
Patient 1 (**A**–**E**) and Patient 2 (**F**). The patients’ bone ages (on the left side with capital letter L for each pair **A**–**F**) compared to male and female standards (on the right side of each pair **A**–**F**) showing brachydactyly type E (brachymetacarpia) with shortening of metacarpal bones (red arrows). (**A**) Male patient’s chronological age: 11 years; bone age: 13–14 years (left), compared with the male standard for age 14. (**B**) Patient’s chronological age: 11 years and 11 months, bone age: 14 years, compared with the male standard for age 14. (**C**) Patient’s chronological age: 12 years and 8 months, bone age: 14 years, compared with the male standard for age 14. (**D**) Patient’s chronological age: 13 years and 4 months, bone age: 15 years, compared with the male standard for age 15. (**E**) Patient’s chronological age: 15 years, bone age: 17 years, compared with the male standard for age 17. (**F**) Female patient’s chronological and bone age: 3 years and 6 months, compared to female standard for this age. Legend: CA—chronological age expressed in years, BA—bone age expressed in years. Greulich–Pyle method of assessment of bone age was applied.

Skeletal maturation was assessed using the Greulich–Pyle comparative method [32]. A radiological evaluation showed advanced bone age, progressing from 14 years at a chronological age of 11 years to 17 years at 15 years of age (Table 1, Figure 2 and Figure 3). During the assessment of the lengths of the phalanges and metacarpal bones, a tendency toward the shortening of the metacarpal bones was observed, which may indicate type E brachydactyly (brachymetacarpia) (Figure 3). A longitudinal follow-up between 2021 and 2025 demonstrated progressive pubertal development, with Tanner stage advancement from II to IV, an increase in testicular volume from 3–4 mL to 15 mL, and the appropriate timing of pubarche and axillarche (Table 1). An endocrine evaluation revealed normal thyroid function throughout follow-up (Table 1). Basal gonadotropin concentrations increased appropriately with pubertal maturation (LH from 1.6 to 2.2 mIU/mL, FSH from 2.0 to 3.6 mIU/mL), and serum testosterone rose from 0.66 to 2.6 ng/mL. Serum insulin-like growth factor 1 (IGF-1) concentrations ranged from 354 to 438 ng/mL and were within the normal range. Overall, the hormonal profile and growth above the 3rd centile on growth charts as seen on Figure 2 did not indicate a growth hormone deficiency or precocious puberty, and the patient was not referred to further GH and GnRH stimulation tests and no growth-promoting therapy was applied.

Relevant comorbidities included a thyroglossal duct cyst excised at 4 years of age and a benign solitary colonic polyp removed surgically. A histopathological examination demonstrated inflammatory changes, initially raising suspicion for inflammatory bowel disease; however, the immunological and serological testing was negative, and no systemic inflammatory disorder was confirmed. The patient had no history of chronic illness, nutritional deficiency, or glucocorticoid exposure.

Due to short stature with advanced bone age and facial dysmorphic features, genetic testing was performed. Genetic analysis revealed a novel heterozygous frameshift variant, c.5677_5684del (p.Glu1893TrpfsTer8), in exon 12 of the *ACAN* gene in the patient and his mother.

### 2.2. Patient 2

**Figure 4:**
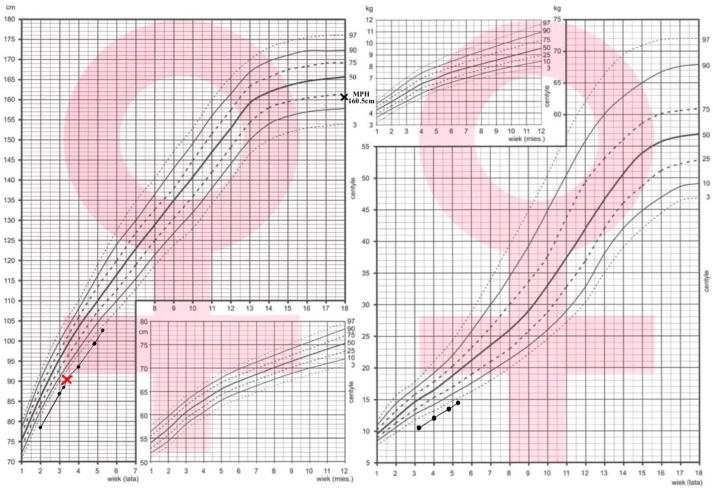
Growth chart of Patient 2. The Polish national charts were obtained from the following website: https://www.mp.pl/pediatria/praktyka-kliniczna/procedury/13848,ocena-rozwoju-somatycznego-dzieci-i-mlodziezy,1 (accessed on 12 December 2025, access after registration to Medycyna Praktyczna). Black dots present the measurements of height/weight. Red cross corresponds to bone age assessment.

The female patient, currently 5.5 years old, has been under endocrinological follow-up since the age of 2 years because of growth failure and poor weight gain. Since birth, her length has remained below the 3rd percentile (Figure 4). At presentation, her height was 78.7 cm, the H SDS was (–) 2.9 SDS, her height age corresponded to 15 months, and her body weight was approximately 10% below the expected value for her height. An anthropometric assessment demonstrated a slightly increased arm span–to–height ratio (AS/H = 1.1), exceeding age- and sex-specific reference values [31]. Her family history was unremarkable. Both parents were healthy. No relatives were reported to have growth failure or skeletal abnormalities. The mother’s height was 155 cm, whereas the father’s height was 178 cm, yielding a mid-parental height (MPH) of 160.5 cm, corresponding to (–) 0.9 SDS.

The patient was born from the first pregnancy at 39 weeks of gestation by cesarean section. The birth weight was 2830 g (−1.56 SDS), and the birth length was 51 cm. The Apgar scores were 10 at both 1 and 5 min. The perinatal period was uneventful. Psychomotor development was within normal limits, with independent sitting achieved at 8 months and walking at 12 months. From infancy, feeding difficulties were noted, including an aversion to solid foods and marked food selectivity, resulting in suboptimal weight gain. A gastroenterological evaluation was performed, and celiac disease was excluded.

Endocrine investigations demonstrated normal thyroid function and a normal insulin-like growth factor 1 (IGF-1) concentration (130.1 ng/mL at 4.5 years of age). Growth hormone deficiency was excluded, with a peak growth hormone level of 11.15 ng/mL during a glucagon stimulation test. A bone age assessment at 3 years and 6 months, based on an X-ray of the left wrist, revealed a bone age consistent with the chronological age (Figure 3F). Magnetic resonance imaging of the pituitary gland showed no abnormalities.

Due to the child’s short stature and an unfavorable bone age consistent with her chronological age, in the presence of normal endocrine findings and subtle dysmorphic features (including a small, receding mandible and down-slanting palpebral fissures), genetic testing was pursued. A conventional cytogenetic analysis revealed a normal female karyotype (46, XX). Copy number variation analysis using next-generation sequencing (NGS) identified a heterozygous deletion encompassing exons 15–19 of the *ACAN* gene. This variant has not been previously reported in the ClinVar database; however, the deletion of five out of nineteen exons is predicted to result in a truncated aggrecan protein with the loss of normal function and is therefore considered the most likely molecular cause of the patient’s growth impairment.

## 3. Genetic Reports

### 3.1. DNA Sequencing and Bioinformatic Analysis

Genomic DNA was extracted from peripheral blood samples from both patients. Two NGS strategies were applied depending on the diagnostic indication: whole-exome sequencing (WES) for Patient 1 and targeted multigene panel sequencing for Patient 2.

### 3.2. Patient 1—Whole-Exome Sequencing (WES)

Whole-exome sequencing was performed using the Twist Human Core Exome Plus Kit (Twist Bioscience, South San Francisco, CA, USA). The enriched DNA libraries were sequenced using the Illumina NovaSeq 6000 instrument (Illumina, Inc., San Diego, CA, USA). All laboratory and sequencing procedures were carried out by CeGaT GmbH (Tübingen, Germany). Raw sequencing reads were mapped to the reference human genome using BWA-MEM2 v2.2.1 [33]. Duplicate reads were removed with Picard 2.18.2-SNAPSHOT (Broad Institute); variants were called using GATK HaplotypeCaller (gatk-4.2.6.1), and annotated with VEP version 105 and Samtools (v1.18) software. The in silico prediction of variant pathogenicity was carried out using Alamut Visual Plus 1.7.2 (SOPHiA GENETICS), Franklin (Genoox), GeneBe, and additional computational prediction tools. The population frequency data were retrieved from dbSNP [34] and gnomAD.

**Figure 5:**
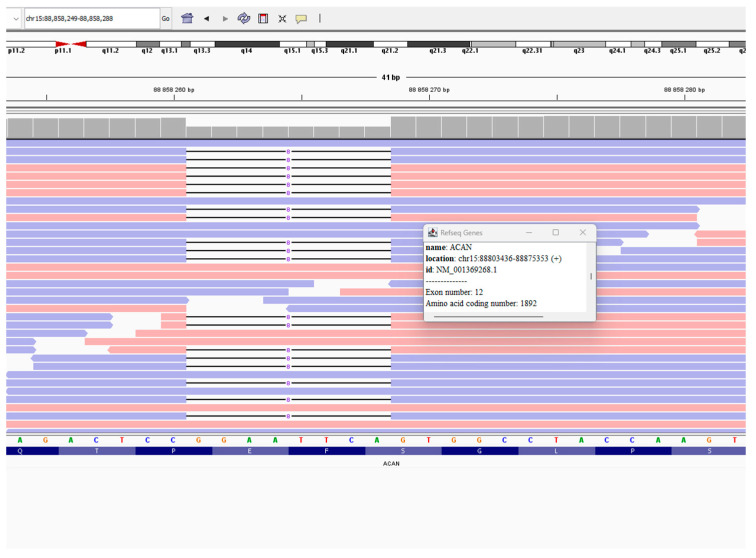
Integrative Genomics Viewer (IGV) visualization of the heterozygous ACAN frameshift variant NM_001369268.1:c.5677_5684del (p.Glu1893TrpfsTer8). The eight-nucleotide deletion is present in a proportion of aligned reads consistent with heterozygosity and results in a frameshift introducing premature stop codon eight amino acids downstream.

The detected genotype of the Patient 1 is NM_001369268.1(ACAN):c.5677_5684del (p.Glu1893TrpfsTer8) in the heterozygous state (Figure 5) The evidence supporting variant pathogenicity according to the ACMG-AMP guidelines includes PVS1 (pathogenic, very strong) and PM2 (pathogenic, moderate).

**Figure 6:**
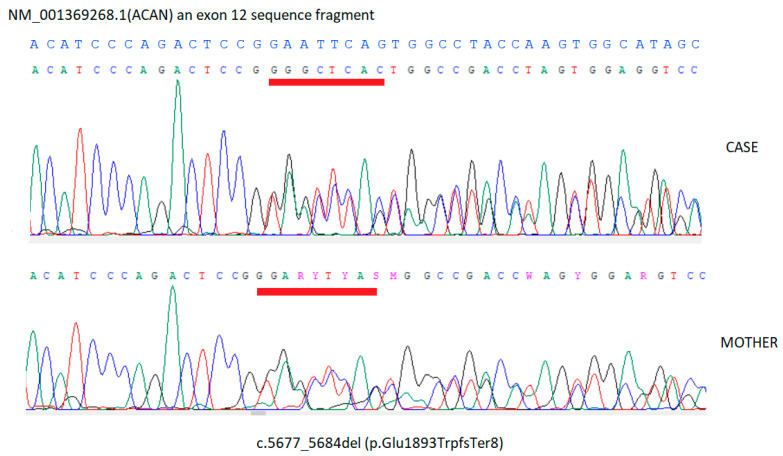
Sanger sequencing chromatograms of the heterozygous NM_001369268.1(*ACAN*):c.5677_5684del (p.Glu1893TrpfsTer8) variant in Patient 1 and his mother.

The detected variant was confirmed by Sanger DNA sequencing performed on a second, independent blood sample (Figure 6). The patient’s mother’s blood sample was also analyzed. Genetic testing for the patient’s father was not performed due to the lack of availability of genetic material for testing. The analysis confirmed the hereditary nature of the detected variant, indicating maternal inheritance (Figure 6).

### 3.3. Patient 2—Targeted Multigene Panel Sequencing

Targeted Next-Generation Sequencing (NGS) was performed using a custom multigene panel including the following genes: *ACAN*, *ANKRD11*, *ARCN1*, *ARID1A*, *ARID1B*, *ARID2*, *ARSB*, *ARX*, *ASXL1*, *ASXL3*, *ATM*, *ATR*, *ATRX*, *BLM*, *BMP2*, *BRAF*, *BTK*, *CBL*, *CCDC8*, *CDC45*, *CDC6*, *CDT1*, *CHD7*, *CREBBP*, *CRIPT*, *CUL7*, *DHCR7*, *DPF2*, *EP300*, *FANCA*, *FANCC*, *FANCF*, *FANCG*, *FGD1*, *FGFR3*, *GHR*, *GHRHR*, *GHSR*, *GMNN*, *GNAS*, *GSC*, *HDAC8*, *HMGA2*, *HRAS*, *IGF1*, *IGF2*, *KANSL1*, *KDM6A*, *KMT2D*, *KRAS*, *LZTR1*, *MAGEL2*, *MAP2K1*, *MAP2K2*, *MCM5*, *MED12*, *NBN*, *NIPBL*, *NRAS*, *OBSL1*, *ORC1*, *ORC4*, *ORC6*, *PHF6*, *PIGG*, *PLAG1*, *POGZ*, *PTPN11*, *RAD21*, *RAF1*, *RIT1*, *RPS6KA3*, *SETBP1*, *SHOC2*, *SHOX*, *SLX4*, *SMARCA2*, *SMARCA4*, *SMARCB1*, *SMARCE1*, *SMC1A*, *SMC3*, *SOS1*, *SOS2*, *SOX11*, *SOX3*, *SPRED1*, *SRCAP*, *THOC2*, *TRIM37*, *UBE2T*, *USP9X*, *VPS13B*, *ZBTB18*, and *ZEB2.* Library preparation was conducted using the SureSelectXT Custom kit (Agilent Technologies, Santa Clara, CA, USA). The sample was sequenced on the Illumina NovaSeq 6000 platform (Illumina, San Diego, CA, USA). Demultiplexing was performed with Illumina’s bcl2fastq2 v2.19.0. Adapter trimming was performed with Skewer v0.2.9 [35]. Reads were aligned to the GRCh37/hg19 human reference genome using BWA-MEM [36]. Duplicate reads were removed using Picard 2.18.2. Variant calling was performed with GATK HaplotypeCaller v4.0.3.0 [37,38] and FreeBayes v1.2.0-2-g29c4002 [39]. Variants were annotated using the following resources: VEP97 [40], including SIFT and PolyPhen-2, dbNSFPv4.0 [41] (MutationAssessor, MutationTaster, DANN, and FATHMM), ESP6500, gnomAD, dbSNP [34], ClinVar, and 1000 Genomes [42].

A copy number variation (CNV) analysis was performed by examining the normalized exon-level coverage profiles for all genes included in the panel. For each sample (BAM file), the single-base coverage across all exons was calculated and normalized by the total number of mapped reads. Significant deviations in the normalized exon coverage were interpreted as potential exon-level deletions or duplications.

**Figure 7:**
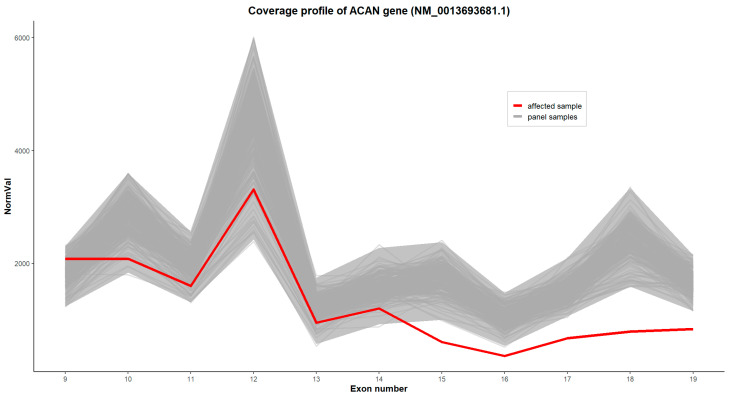
The normalized coverage profile for *ACAN* gene (transcript NM_0013693681.1) across the analyzed exons. The *x*-axis contains exon numbers, and the *y*-axis shows normalized coverage values scaled to an arbitrary unit. The gray line represents the range of coverage in the reference samples from the panel, while the red line represents the values obtained for the accession under study. The visible decrease in signal corresponds to a deletion spanning chr15: 88,871,381_88,744,481 (Human genome reference: GRCh38).

A large deletion of 5 exons was identified in the *ACAN* gene. We present the image from the in-house CNV analysis, showing a deletion of exons 15–19 of the *ACAN* gene (chr15: 88,871,381_88,744,481), according to the reference sequence NM_0013693681.1 in Figure 7. The evidence supporting variant pathogenicity according to the ACMG guidelines includes 2C_1 (+0.9, Partial overlap with the 5’ end of an established HI/LOF-sensitive gene).

Verification of the detected deletion using methods dedicated to CNV analysis (e.g., array comparative genomic hybridization (aCGH) or MLPA: probes targeting the ACAN gene are not available) was not performed. Moreover, segregation analysis within the patient’s family was not carried out due to the family’s refusal to consent to further genetic testing. For this reason, the genetic diagnostic process for the patient was also discontinued, while acknowledging the limitations of the applied method (targeted NGS multigene panel). Although this approach allows for the efficient evaluation of genes known to be associated with short stature, it restricts the analysis to a predefined set of genes and, therefore, does not enable the identification of pathogenic variants in genes not included in the panel. Furthermore, the applied method primarily covers the coding regions and exon–intron boundaries; thus, variants located in deep intronic regions, regulatory elements, or other non-coding sequences were not analyzed as well. Despite the limitations of the applied genetic diagnostic method, the obtained result correlated with the clinical indications for testing and allowed for the confirmation of a large deletion within the *ACAN* gene, which could be associated with the patient’s phenotype

### 3.4. Variant Interpretation

For both patients, the pathogenicity of identified variants was assessed according to the 2015 American College of Medical Genetics and Genomics and the Association for Molecular Pathology (ACMG-AMP) guidelines and using the bioinformatics tools described above [43].

## 4. Discussion

The two patients presented in this report illustrate both the phenotypic breadth of aggrecanopathy and the practical diagnostic challenges that continue to surround *ACAN*-related short stature. In both children, short stature occurred in the setting of a normal endocrine evaluation and the absence of chronic systemic disease; however, genetic testing clearly identified the causative abnormalities in the *ACAN* gene. In the first patient, we detected a novel heterozygous truncating variant, c.5677_5684del (p.Glu1893TrpfsTer8), in exon 12; in the second, copy number analysis revealed a heterozygous deletion encompassing exons 15–19. These findings are relevant not only because they establish the molecular diagnosis, but also because they expand the mutational spectrum of aggrecanopathy. In particular, no PubMed-indexed, peer-reviewed cases of deletions involving *ACAN* exons 15–19 were identified. Clinically, both patients further broaden the dysmorphologic spectrum of the disorder. The facial pattern observed in our patients, including ptosis, hypertelorism, down-slanting palpebral fissures, mandibular retrognathia, and fleshy auricles, has not been emphasized in prior reports and may provide an additional diagnostic clue in children who might otherwise be classified as having idiopathic or familial short stature.

The prevalence of *ACAN* variants in unselected short stature cohorts is modest; however, in clinically enriched groups, the diagnostic yield increases substantially [3,9,28,44,45,46,47]. Lin et al. identified pathogenic *ACAN* variants in 1.2% of 1005 short children overall, but the frequency increased to 14.3% among children with advanced bone age and to 35.7% among those with both advanced bone age and a family history of short stature [9]. Similarly, van der Steen et al. identified *ACAN* variants in 4 of 290 short children born small for their gestational age, indicating that *ACAN* deficiency should also be considered in the differential diagnosis of persistent post-SGA short stature, particularly when accompanied by advanced bone age or additional skeletal features [28]. Taken together, these data indicate that aggrecanopathy is not merely an exceptional diagnosis, but rather a recurrent and clinically relevant cause of short stature in selected phenotypic contexts [28,47,48]. These observations are consistent with the increasingly recognized view that pathogenic *ACAN* variants represent an important monogenic cause of growth failure and are likely underdiagnosed in routine pediatric endocrinology practice [14,15,44,45,46,47,48,49,50,51,52,53,54,55,56,57,58,59,60,61,62,63,64,65,66,67,68,69,70,71,72,73,74,75,76,77,78,79,80,81,82].

For a comparison with Patient 1, we systematically identified and compiled cases reported in the literature involving pathogenic variants in exon 12, which are summarized in Table 2.

The findings observed in our patient with a truncating pathogenic variant in exon 12 can be coherently integrated with both cohort-level data and current knowledge on *ACAN* structure–function relationships [1,3,7,9,10,11,17,23,25,28,29,30,47,54,56,63,64,67,68,69,70,72,73,75,78]. Exon 12 represents a mutational hotspot within *ACAN*, accounting for approximately 19% of reported pathogenic variants [9,69]. This exon encodes the chondroitin sulfate attachment domains (CS1 and CS2) and, together with exon 11, contributes to the keratan sulfate (KS) attachment region as shown on Figure 1 [18,19,20,21]. The sulfation of these domains and their aggregation with hyaluronan generate a high fixed negative charge density, which is essential for cartilage hydration and resistance to mechanical load [21]. The disruption of this region is therefore expected to have profound effects on both the growth plate and articular cartilage biology.

In this context, the phenotype observed in our patient aligns with the clinical spectrum reported in published cases with pathogenic variants in exon 12 (Table 2) [1,3,7,9,10,11,17,23,25,28,29,30,47,54,56,63,64,67,68,69,70,72,73,75,78]. In a cohort of 41 individuals, the mean height SDS was −2.82, with short stature (≤−2 SDS) present in 83% and severe short stature (≤−3 SDS) in 31%, consistent with previously described *ACAN* cohorts [67]. Our patient’s growth impairment falls within this range, supporting the notion that exon 12 variants are consistently associated with a clinically significant linear growth deficiency. Similarly, the frequency of skeletal and craniofacial features in that cohort—brachydactyly (~30%), midfacial hypoplasia (~20%), and frontal bossing (~15%)—parallels the broader phenotypic spectrum described in the literature, although our patient additionally exhibited subtle but distinctive dysmorphic features, including mild bilateral ptosis, hypertelorism, down-slanting palpebral fissures, fleshy auricles, and retrognathia [30,67]. These findings may represent an extension of the craniofacial phenotype associated with an *ACAN* deficiency [28,30,47].

Notably, our patient also demonstrated a mild limitation of elbow extension and flat valgus feet, raising concern for early osteoarticular involvement. Although degenerative joint manifestations were reported in only ~14–17% of individuals in the exon 12 cohort-likely reflecting age-dependent penetrance, these features are well-recognized to emerge later in life and may be underrepresented in pediatric cohorts [30,67]. In this regard, these observations may support the concept that variants affecting the KS and CS domains are predisposed not only to growth plate dysfunction but also to early articular cartilage vulnerability.

Advanced bone age was reported in ~45–60% of cases in the cohort, consistent with more recent data indicating that this feature is frequent but not universal [3,47,67].

Given the limited number of reported deletions involving *ACAN* (particularly exons 15–19), all available cases from the recent literature were additionally compiled in Table S1 to provide a broader overview of genotype–phenotype correlations at the gene level.

Across the aggregated cases and cohorts (Table S1), short stature emerged as the dominant and most penetrant phenotype associated with *ACAN* variants. Short stature was present in a great majority of evaluable individuals, affecting approximately 85–90%, whereas severe short stature was observed in roughly 55–65%. The mean pretreatment height SDS ranged from approximately −3.0 to −3.3, consistent with a predominantly moderate-to-severe linear growth deficit. Among the accompanying skeletal and dysmorphic findings, brachydactyly or broad digits were present in about 45–55% of reported cases, frontal bossing in approximately 30–40%, and advanced bone age in about 60–70%, making accelerated skeletal maturation one of the most recurrent associated features.

These aggregated observations are consistent with prior reports, thereby reinforcing the concept that short stature represents the core phenotypic feature of aggrecanopathies, while additional manifestations show variable expressivity [3,14,28,29,30,31,32,33,34,35,36,37,38,39,40,41,42,43,44,45,46,47,48,49,50,51,52,53,54,55,56,57,58,59,60,61,62,63,64,65,66,67,68,69,70,71,72,73,74,75,76,77,78,79,80,81,82]. Similarly, brachydactyly, broad thumbs or great toes, midface hypoplasia, frontal bossing, and other minor skeletal features occur with sufficient frequency to be diagnostically informative, although none is universally present [9,11,28,44,47,48,50,54,70,79,80,81,82]. Mild disproportion, often reflected by an increased sitting height–to–height ratio or arm span–to–height ratio, may be subtle in early childhood and become more apparent with age [9,11,28,47,48,80,81,82]. Joint manifestations, including osteochondritis dissecans, early osteoarthritis, and intervertebral disc disease, also exhibit age dependence, becoming more prominent in adolescence and adulthood [25,44,54]. Advanced bone age, while often regarded as a hallmark of *ACAN*-related short stature, is increasingly recognized as neither constant nor obligatory [3,9,28,47,48,67,69]. This has important clinical implications, as the absence of bone age acceleration does not exclude an *ACAN* deficiency.

Accordingly, both our aggregated analysis and the existing literature emphasize that diagnostic suspicion should not rely on any single feature, but rather on a recurring constellation of findings, including short stature, familial clustering, subtle disproportion, brachydactyly or broad digits, midface hypoplasia, and either advanced bone age or an atypical growth trajectory [28,46,47,48].

Our cases are especially informative in this regard because they also illustrate the natural history of a missed diagnosis. The older boy exemplifies a pattern repeatedly described in the literature: growth that may appear relatively preserved in childhood, followed by accelerated skeletal maturation with the onset of puberty, a rapid narrowing of the growth window, and early epiphyseal fusion [3,14,15,28,46,47,48]. In retrospect, the absence of a classical endocrine pathology in this patient was misleading. Because his auxological and pubertal features did not meet the national criteria for the initiation of growth hormone treatment or for therapeutic pubertal suppression, no growth-modifying intervention was undertaken at a time when meaningful residual growth potential still existed. Once the molecular diagnosis was established, it became evident that the key pathology was not endocrine deficiency but a primary disorder of the growth plate. This distinction is crucial, because conventional endocrine algorithms may fail precisely in the patients who would benefit most from earlier genotype-informed treatment.

This clinical pattern can now be more coherently interpreted in light of the experimental data [49]. In particular, the studies by Bendre et al. extend the understanding of aggrecanopathies beyond descriptive clinical observations by providing a biologically plausible mechanistic framework for both the growth disturbance and the variable response to therapy [49]. In *Acan*+/− mouse models, postnatal growth failure was not primarily attributable to impaired chondrocyte proliferation [49]. Rather, proliferative activity was relatively preserved, whereas hypertrophic differentiation and extracellular matrix expansion were disrupted [49]. More specifically, a reduced aggrecan expression resulted in impaired extracellular matrix production and the defective maturation of hypertrophic chondrocytes, accompanied by the suppression of Akt signaling in the prehypertrophic and hypertrophic zones [49].

This distinction is clinically and biologically relevant. Linear growth depends not only on the number of proliferating chondrocytes, but also on their capacity to undergo appropriate hypertrophy and matrix expansion. Accordingly, when proliferation is relatively maintained but hypertrophic maturation is compromised, children may initially demonstrate near-normal growth velocities, yet fail to achieve adequate longitudinal bone growth over time. This imbalance becomes particularly evident during puberty, when accelerated growth plate senescence further limits growth potential. Within this framework, both our observations and prior reports can be reconciled, supporting the concept that *ACAN*-related short stature represents a cartilage-intrinsic disorder rather than a primary endocrine defect, thereby explaining why systemic hormonal therapies yield only partial and variable efficacy [14,15,28,46,47,48,49].

The increased arm span-to-height ratio and sitting height-to-height ratio observed in these patients can be similarly interpreted through this mechanistic lens, reflecting the segment-specific vulnerability of growth plates [49]. The weight-bearing physes of the lower limbs, particularly at the distal femur, appear more susceptible to aggrecan deficiency due to higher mechanical load and rapid growth dynamics, resulting in the preferential shortening of the legs [49]. In contrast, the relatively preserved growth of the upper limbs maintains the arm span, leading to a disproportionate increase in the arm span-to-height ratio [49].

The clinical literature is largely concordant with this biological framework [46,47]. Genotype–phenotype analyses consistently indicate that truncating variants are associated with a more severe auxologic phenotype than non-truncating variants [46,47]. For example, in the Dutch real-world cohort reported by Renes et al., children harboring truncating variants were significantly shorter at baseline than those with non-truncating variants, with median height SDS values of −2.8 versus −2.1 [47]. Similarly, Wu et al. demonstrated that truncating variants were linked not only to more pronounced short stature but also to more advanced bone age [46].

Within this integrated genotype–mechanism–phenotype framework, the recombinant human growth hormone remains the principal growth-promoting therapy; however, its expected benefit should be interpreted with appropriate caution [46]. As demonstrated by Bendre et al., GH therapy may exert its effect in aggrecanopathy by partially compensating for the cartilage-intrinsic defect in IGF-I–Akt signaling [49]. By increasing the circulating IGF-I levels, GH enhances the activation of the PI3K/Akt pathway within the growth plate, thereby promoting hypertrophic chondrocyte differentiation and cellular hypertrophy—processes that are specifically impaired in *ACAN* haploinsufficiency [49]. Importantly, both experimental data and clinical observations suggest that this effect is likely to be most beneficial when therapy is initiated early, before advanced bone age and growth plate senescence significantly constrain the residual growth potential [49].

The accumulated evidence indicates that rhGH can be beneficial in aggrecanopathy [28,45,46,47,48]. Muthuvel et al., in an open-label prospective study of prepubertal children with heterozygous *ACAN* variants, reported an increase in the median height velocity from 5.2 to 8.3 cm/year and a median height gain of +0.62 SDS after one year, without the inappropriate advancement of bone age [45]. Sun et al. observed an improvement in the mean height from −2.89 ± 0.68 SDS to −1.91 ± 0.93 SDS over approximately 1.85 years [56]. In the Renes et al. study, prepubertal children gained 1.0 SDS over three years, whereas pubertal children mainly stabilized their height SDS rather than improving it [47]. In the Gkourogianni et al. cohort during growth hormone therapy, the height SDS increased modestly, by approximately +0.4 in the first year, +0.7 after two years, and +1.0 after three years [7]. In the Trigui et al. study, prepubertal children gained 1.6 SDS over five years, whereas pubertal children gained 0.4 SDS [3]. The newer family study by Shalev-Goldman et al. documented a rise in the height SDS from −3.3 ± 1.0 to −2.0 ± 1.1, corresponding to a mean gain of 1.3 ± 0.3 SDS, with the greatest response occurring in the first treatment year [48]. These observations are also broadly consistent with Tang et al., who found an overall significant improvement in the height SDS, although with substantial interindividual variability [67].

What emerges most clearly from these studies is not simply that GH works, but that timing is critical. The best responses are consistently seen in children treated before puberty and before the marked advancement of bone age [10,28,46,47,48]. Wu et al. concluded that children starting rhGH before the age of 10 years responded more favorably, whereas later treatment was less effective, as observed also in the cohort of Stavber et al. [10,46]. Renes et al. likewise demonstrated a clear difference between prepubertal and pubertal patients, and Shalev-Goldman et al. emphasized that the greatest acceleration in growth velocity occurred early in treatment, before progressive pubertal maturation curtailed the remaining growth window [47,48]. In practical terms, therefore, the most defensible conclusion is that GH therapy in *ACAN*-related short stature should be initiated as soon as the diagnosis is established and while substantial prepubertal growth potential remains, ideally well before 10 years of age whenever possible.

This conclusion, however, exposes a major translational problem. In some countries, including our own, children may not qualify for reimbursed GH therapy if their height remains above the 3rd centile, even when they carry a pathogenic *ACAN* variant and have a biologically unfavorable prognosis. Similarly, there is no dedicated national protocol for suppressing puberty in 10- to 11.5-year-old children with genetically confirmed aggrecanopathy and accelerated skeletal maturation. As a result, children with a recognized growth-plate disorder may remain ineligible for intervention simply because they do not meet the criteria designed for other conditions, such as GH deficiency, SGA, Turner syndrome, Prader–Willi syndrome, or central precocious puberty. Our older patient illustrates exactly this gap between molecular diagnosis and therapeutic accessibility. From a clinical standpoint, this is problematic, because, by the time height falls below conventional thresholds or bone age acceleration becomes extreme, the optimal window for intervention may already have been lost.

The role of puberty modulation should therefore be discussed in this specific context. In *ACAN* deficiency, puberty may be physiologically timed yet biologically disadvantageous, because estrogen-mediated growth plate senescence can sharply accelerate the already compromised process of longitudinal growth. For this reason, several authors have considered combining GH with gonadotropin-releasing hormone analogues in selected patients [28,46,47,48,67]. The most compelling clinical support comes from van der Steen et al., who treated four short SGA children with *ACAN* variants using GH plus two years of GnRHa from pubertal onset and a reported improvement in the height SDS by approximately 0.7 SDS; at adult height, one girl and one boy were 5.2 cm and 8.0 cm taller, respectively, than their same-sex affected parent [28]. Shalev-Goldman et al. also introduced GnRHa at pubertal onset in children entering puberty between 10 and 11.6 years, although they appropriately emphasized that the incremental contribution of GnRHa remains uncertain [48]. Renes et al. and Tang et al. likewise suggest that a combined treatment may help in selected situations, but not uniformly [47,67]. The most balanced interpretation, therefore, is that GnRHa should not be considered the routine therapy for all patients with *ACAN* variants, but may be reasonable at the beginning of puberty, especially around 10–11.5 years, in children with rapidly advancing bone age and a high risk of losing their residual height potential [28,46,47,48,57,59]. Unfortunately, as noted above, such an approach is not supported by the national treatment pathways in many healthcare systems.

Aromatase inhibitors are even less well-established. van der Steen et al. described delayed skeletal maturation in boys treated with GnRHa followed by letrozole, with a reassuring short-term bone mineral density, but adult-height data remained limited [28]. Wu et al. have also suggested that aromatase inhibitors may be considered in pubertal boys with advanced bone age, particularly as an adjunct when the growth potential is rapidly diminishing [46]. Nevertheless, the evidence is too sparse to justify routine use, and these agents should presently be viewed as individualized, highly selected options rather than standard care.

Any therapeutic discussion in aggrecanopathy should also extend beyond stature itself. The musculoskeletal phenotype is clinically important, and treatment aims should include the preservation of joint function and reduction in long-term orthopedic morbidity [80,81]. Sentchordi-Montané et al. emphasized that no disease-specific therapy currently exists and that management should include attention to joint protection, weight control, and appropriate physical activity while minimizing excessive joint loading [80,81]. This caution is particularly relevant because *ACAN* deficiency predisposes one to osteochondritis dissecans and early osteoarthritis. Although GH has generally been well-tolerated, Ochoa et al. described a boy with a pathogenic *ACAN* variant in exon 12, who developed painful multifocal osteochondritis dissecans shortly after the initiation of GH therapy [73]. Although the causality remains uncertain, this report supports careful clinical surveillance during treatment. In Renes et al., scoliosis was noted in 2 of 36 children during GH treatment, although it remains unclear whether this reflected the therapy, the underlying disease progression, or both [47]. Accordingly, endocrine treatment in *ACAN* deficiency should always be accompanied by musculoskeletal follow-up.

Another possible height-restoring option is surgical limb lengthening. This should probably be mentioned, but with substantial caution. No robust disease-specific data exist for patients with pathogenic *ACAN* variants. Evidence comes largely from adult cosmetic limb-lengthening series [83]. In the meta-analysis by Giorgino et al., the mean height gain was approximately 67 mm, patient satisfaction ranged from 88.8% to 98%, and psychological outcomes often improved, including body image and self-esteem [83]. At the same time, the complication burden was substantial, including joint and tendon problems, infections, bone-healing complications, device-related failures, and neurologic or vascular events, with prolonged rehabilitation. In the setting of aggrecanopathy, where the cartilage biology is intrinsically abnormal and musculoskeletal vulnerability may already be present, these concerns become even more relevant [18,83]. Limb elongation may therefore be regarded only as a highly individualized rescue option in exceptional cases, most reasonably after growth completion and after careful multidisciplinary counseling [83]. It cannot at present be viewed as a standard or first-line treatment for *ACAN*-related short stature.

Collectively, our cases and the existing literature support a coherent and clinically applicable framework for diagnostic reasoning. First, *ACAN* deficiency should be suspected early in children with unexplained short stature, particularly when there is familial clustering, advanced bone age, subtle disproportion, brachydactyly, broad great toes, or characteristic facial features [28,47]. Second, once the diagnosis is established, the child should not be managed solely according to conventional endocrine frameworks, because the disorder is fundamentally a disease of growth plate cartilage [49]. Third, if growth-promoting therapy is being considered, the evidence strongly favors intervention early, preferably in the prepubertal years and ideally before 10 years of age [48]. Fourth, at the onset of puberty, especially around 10–11.5 years in children with clearly accelerated skeletal maturation, GnRHa may be considered in selected cases, although the evidence remains incomplete and national access is often limited. Finally, treatment decisions should always incorporate the broader musculoskeletal risks of aggrecanopathy and the reality that the current reimbursement or protocol-based systems may fail to serve these patients adequately.

In summary, our study broadens both the molecular and dysmorphological spectrum of aggrecanopathy and reinforces the need for early molecular diagnosis in children with an apparently idiopathic short stature. The clinical course of our older patient illustrates how the reliance on the standard endocrine eligibility criteria may result in a missed therapeutic window in a child with a genetically determined growth-plate disorder. At the same time, mechanistic data from Bendre et al. provide a coherent explanation for why these children can initially maintain relatively preserved growth despite fundamentally impaired hypertrophic chondrocyte maturation, and why treatment response depends so strongly on timing [49]. Altogether, these observations support a shift from purely phenotype-based or endocrine-based algorithms toward earlier genotype-informed management, with an individualized consideration of GH, selected pubertal modulation, longitudinal orthopedic surveillance, and—in only exceptional situations—surgical height restoration.

## Figures and Tables

**Table 1:** Follow-up visits of Patient 1.

Chronological Age (Years)	11	12.8	13.4	15
Bone age (years)	14	14	15	17
		Tanner scale		
Testes volume R, L	3, 4	8, 8	10, 10	15, 15
Axillarche	-	-	-	+
Pubarche	I	I	II	V
Male Genitalia Tanner Stages (G)	I/II	II	III	IV
		Hormonal data		
IGF-1 (ng/mL)		354.2	433.8	302.8
LH (mlU/mL)		1.62	2.17	
FSH (mlU/mL)		2.2	3.6	
Testosterone (ng/mL)		0.6–2.70	2.58	
TSH (ulU/mL)	1.3	0.9	0.96	0.8
fT4 (pmol/L)	1.33	14.3	14.3	16.3

**Table 2:** Genotype and clinical information in 41 patients with pathogenic variants in exon 12 of *ACAN* gene. Abbreviations: SGA—small for gestational age, BA—bone age, CA—chronological age, ABA—advanced bone age, NBA—normal bone age, MFH—midfacial hypoplasia, PF—prominent forehead, FB—frontal bossing, FNB—flat nasal bridge, B—brachydactyly, IDD—intervertebral disc diseases, SS—short stature, OA—early-onset osteoarthritis, OCD—osteochondritis dissecans, SEDK—spondyloepiphyseal dysplasia, Kimberley type, SEMD—spondyloepimetaphyseal dysplasia, SN—short neck, GH—growth hormone, GnRH—gonadotropin-releasing hormone, NA—non available, ND—no data, Ref.—reference, H SDS—height standard deviation score, TH—target height.

Case ID	Gene Variant (cDNA)	Protein	Sex	Age (yr)	H SDS	TH SDS	Cranio- Facial Features	Skeletal Features	Bone Age	Other Findings	Therapy Outcome H SDS Gain	Ref.
1	c.2369C>G	p.Ser790*	M	14.5	−2.2	−0.6	FB, hypertelorism	B	ABA 16.5 yr	Hyperlordosis	GH therapy started, outcome not known	Sentchordi-Montané et al. 2018 [11]
2	c.2218A>T	p.Thr740Ser	M	3	−3.2	ND	FB, MFH	B	BA/CA = 0	No	No therapy	Sentchordi-Montané et al. 2018 [11]
3	c.2535_2536insTTCA	p.Pro846Phefs*9	M	4.9	−2.5	−0.16	No	No	BA = CA	Familial SS	No therapy	Hattori et al. 2017 [72]
4	c.4259A>G	p.Glu1420Gly	F	6.2	−2.48	−0.51	No	No	BA delayed	no	No therapy	Hattori et al. 2017 [72]
5	c.6530T>C	p.Val2177Ala	F	6.8	−2.26	−0.17	No	No	NA	no	No therapy	Hattori et al. 2017 [72]
6	c.2677delG	p.Gly893AspfsTer52	Fetus	21w + 3d	ND	ND	PF, mild nasal hypoplasia	Isolated shortening of fetal long bones/rhizomelic involvement	Prenatal	First prenatal ACAN skeletal-dysplasia report	No therapy	Toscano et al. 2021 [78]
7	c.3986dupC	p.Gly1330fs*221	ND	ND	ND	ND	No	SEDK	ND	No	No therapy	Gleghorn et al. 2005 [25]
8	c.4138G>T; c.5061T>A	p.Val1380Phe; p.Ser1687Arg	M	45.0	−9.1	ND	No	SEMD	ND	Osteogenesis imperfecta diagnosed at 6 yr	No therapy	Fukuhara et al. 2019 [29]
9	c.4390delG	p.Val1464Ter	F	10.8	−2.5	−2	Macrocephaly, FB	B, broad thumbs	BA = CA	OA, OCD, familial SS with early-onset IDD; Precocious puberty, no GHD	GH +GnRH agonist Gain: +0.2	Mancioppi et al. 2021 [30]
10	c.4390delG	p.Val1464Ter	F	7.6	−2.4	−2	Macrocephaly, FB	B, broad thumbs	BA = CA	OA, OCD, familial SS with early-onset IDD, normal puberty, no GHD	GH + GnRH agonist Gain +0.3	Mancioppi et al. 2021 [30]
11	c.2441C>G	p.Ser814*	M	13	−1.5	−2.4	ND	ND	ABA	ND	No therapy	Trigui et al. 2025 (P3) [3]
12	c.2441C>G	p.Ser814*	M	27	−3.8	NA	ND	Hallux valgus x2	ND	Isolated SS in son	No therapy	Trigui et al. 2025 (P4) [3]
13	c.2515G>T	p.Glu839*	F	8	−2.5	0.5	ND	Rhizomelia	ND	Mother and grandmother with SS and EOA	Gain over 5 years of GH: +0.4	Trigui et al. 2025 (P5) [3]
14	c.4646dupC	p. Ser1550Phefs*12	F	13	−3	−3.5	No	B	ND	Mother with SS and B	No therapy	Trigui et al. 2025 (P9) [3]
15	c.6189_6190dupAC	p. Pro2064Hisfs*29	F	3	−4	−3	MFH	B	ABA	No	No therapy	Trigui et al. 2025 (P10) [3]
16	c.4887delT	p. Phe1629Leufs*33	F	11	−1.5	−2	No	No	ND	Scoliosis	GH treated, no data on outcome	Trigui et al. 2025 (P15) [3]
17	c.4634delT	p.Leu1545Profs*11	F	7.8	−3.6	ND	MFH	Short 4th toe, broad 5th toes	ABA 8.10 yr	Familial SS with early-onset IDD	No data	Uchida et al. 2020 [63]
18	c.6229delG	p.Asp2078Thrfs*14	M	ND	−3.74	ND	No	No	ND	SS	No data	Tang et al. 2024 [67]
19	c.4657G>T	p.Glu1553*	F	ND	−3.2	ND	MFH, FNB	Short thumbs	ABA	Osteoarthritis; familial SS with IDD	Modest	Gkourogianni et al. 2017 [7]
20	c.4762_4765del	p.Gly1588fs*26	M	12.3	−2.7	ND	MFH, posteriorly rotated ears	Broad great toes	ABA 13.3 yr	Hip joint problems	GH +GnRH Gain: +0.1	van der Steen et al. 2017 [28]
21	c.4852C>T	p.Q1618*	M	11.7	−4.0	ND	MFH, retrognathia,	No	BA = CA	OA, IDD, GHD	Modest gain on GH therapy	Tatsi et al. 2017 [17]
22	c.5391delG	p.Gly1797fs*52	M	5.6	−3.2	ND	MFH, FNB	Short thumbs	Markedly ABA	Familial SS, hip joint problems	No data	Quintos et al. 2015 [23]
23	c.5443delC	p.Leu1815fs	M	4.0	−4.4	ND	No	No	BA = CA	No	GH Gain: +1.07	Lin et al. 2021 [9]
24	c.5579delC	p.Gly1861fs	F	9.4	−2.9	ND	No	No	ABA 10.7 yr	No	GH Gain: +0.3	Lin et al. 2021 [9]
25	c.5597C>A	p.Ser1866*	M	ND	−2.0	ND	PF	B	ABA	Short neck, barrel-shaped chest	No therapy	Hauer et al. 2017 [1]
26	c.6142C>G	p.Pro2048Ala	F	12.5	−2.2	−2.1	No	B	BA = CA	No	No therapy	Sentchordi-Montané et al. 2018 [11]
27	c.5391dup	p. Gln1798AlafsTer17	M	13	−2.3	+0,02	No	Multi-sides OCD	ABA (+2 years)	OCD	Side-effect of GH therapy (OCD)	Ochoa et al. 2023 [73]
28	c.5677_5684del	p.Glu1893TrpfsTer8	M	11	−2.0	−0.8	Yes	Joint pain	ABA	Knee pain	Not treated	Own case
29	c.5658delG	p. Phe1887LeufsTer15	M	13	3c	ND	No	Bilateral elbow and right knee osteochondral defects, left tibial osteochondroma	ND	No	Not treated	Denis et al. 2022 [54]
30	c.5658delG	p. Phe1887LeufsTer15	M	13	ND	ND	No	No	ND	SS	Not treated	Denis et al. 2022 [54]
31	c.5658delG	p. Phe1887LeufsTer15	M	Father	−2.7	ND	No	No	ND	SS	Not treated	Denis et al. 2022 [54]
32	c.2367delC	p.Ser790Glnfs*20	F	7.9	+1.08	ND	No	Yes	Precocious puberty ABA	No	GH+ GnRH, poor outcome	Wei et al. 2021 [68]
33	c.2660C>G	p.Ser887Ter	M	2.9	−3.05	ND	Yes	Yes	NBA	No	GH Gain: +1.7	Sun et al. 2022 [56]
34	c.2911G>T	p.Gly971Ter	M	3.4	−3.08	ND	Yes	Yes	NBA	No	GH Gain: +0.62	Sun et al. 2022 [56]
35	c.2266G>C	p.Gly756Arg	M	3	−2.92	ND	MFH, FB, FNB, long philtrum	Short thumbs, short metacarpals	ABA	Short neck, scoliosis, lumbar lordosis, rib valgus	GH Gain: +0.76	Liang et al.2019 [69]
36	c.5239_5248del	p. Gly1747Leufs*3	F	5.4	−2.1	ND	No	No	ABA	No	No therapy	Andrade et al. 2022 [70]
37	c.2367delC	p. Ser790Glnfs*20	F	3	−2.24	ND	No	No	ABA	No	No therapy	Zhao et al. 2023 [75]
38	c.2735_2736delins31	p.Leu912fs	F	4.2	−4.8	−1.8	No	No	DBA	Knees OCD	Gh Good outcome	Renes et al. 2024 [47]
39	c.2735_2736delins31	p.Leu912fs	F	11.9	−2.2	−2.25	No	B	ABA	Sandal gap	GH +GnRH Good outcome	Renes et al. 2024 [47]
40	c.2770G>T	p.Glu924*	M	3.3	−2.6	0.85	FB	B	ABA	Baker’s cysts	GH Good outcome	Renes et al. 2024 [47]
41	c.6404delC	p.Ala2135Aspfs	M	6.9	−1.75	−0.03	MFH	No	ABA 9.9 yr	Disproportionate SS	GH Gain: +1.97	Tatsi et al. 2017 [17]

## Data Availability

The original contributions presented in this study are included in the article/Supplementary Material. Further inquiries can be directed to the corresponding authors.

## Supplementary Materials

The following supporting information can be downloaded at: https://www.mdpi.com/article/10.3390/diseases14040127/s1, Table S1: Genotype–phenotype information based on available data (n = 302 patients with pathogenic ACAN variants).
